# The effects of detraining and training on adipose tissue lipid droplet in obese mice after chronic high-fat diet

**DOI:** 10.1186/s12944-016-0398-x

**Published:** 2017-01-17

**Authors:** Ju Yong Bae, Jinhee Woo, Hee Tae Roh, Yul Hyo Lee, Kangeun Ko, Sunghwun Kang, Ki Ok Shin

**Affiliations:** 1 Laboratory of Exercise Biochemistry, Department of Physical Education, College of Arts and Physical Education, Dong-A University, 37 Nakdong-daero 550 beon-gil, Hadan-dong, Saha-gu, Busan 604-714 Republic of Korea; 2 Laboratory of Exercise physiology, Division of Sport Science, Kangwon National University, 1 Kangwondaehak-gil, Chuncheon-si, Gangwon-do 24341 Republic of Korea

**Keywords:** Exercise, Treadmill exercise, Aerobic exercise, Obesity, Detraining, Abdominal visceral fat

## Abstract

**Background:**

It is well known that exercise promotes lipolysis by stimulating the lipid droplet (LD) signaling pathway. However, few studies have been conducted to examine the effect of detraining with high fat diet (HFD) and training effects after long-term HFD. Here, we investigated the effect of detraining and training on adipose tissue LD pathway in diet-induced obese mice after continuous HFD.

**Methods:**

Seventy male C57BL/6 mice were randomly assigned into a Normal diet + Sedentary group (ND, *n* = 10) or a High-fat diet + Sedentary group (HF, *n* = 50); in the HF group, obesity was induced by a 45% fat chow for six weeks. For the subsequent eight weeks, the HF group was randomly subdivided into an HF (*n* = 30) or an HF + training group (HFT, *n* = 20), and the HFT group was subjected to treadmill training while on an HFD. Following this eight-week period, the HFT group stopped exercising (HFT-DT group, *n* = 10), and the mice in the HF group were randomly subdivided into an HF (*n* = 10) or an HF + training group (HF-T, *n* = 10). After training and detraining, abdominal visceral fat was obtained and analyzed by histological staining and western blot.

**Results:**

Treadmill exercise decreased body weight and fat mass (*P* <0.05), and increased the levels of PKA, perilipin1, CGI-58, ATGL, and HSL (*P* <0.05) after eight weeks of training. Following eight weeks of detraining, the levels of PKA and HSL were decreased (*P* <0.05); however, exercise after chronic HFD increased the levels of PKA, perilipin1, CGI-58, ATGL, and HSL (*P* <0.05), and decreased body weight and fat mass (*P* <0.05).

**Conclusions:**

Regardless of dietary restrictions, exercise is an effective treatment for obesity, owing to the regulation of LD signaling proteins. Moreover, the effects of regular exercise after chronic HFD were similar to those of exercise in the absence of HFD. Therefore, although obesity is induced by chronic HFD, exercise without dietary change is sufficiently effective for obesity treatment regardless of the preceding HFD period.

## Background

Reduced satiety caused by continuous high-fat diet (HFD) intake increases food intake [1]. Moreover, obesity induced by chronic HFD is a risk factor for cardiovascular disease and metabolic disorders, such as dyslipidemia and insulin resistance [2, 3]. Because the prevalence of obesity has increased consistently over the past 30 years [4], an appropriate solution is needed.

Ingested fat is mainly stored in the form of triacylglycerides (TAGs) in lipid droplets (LDs). These TAGs are surrounded by a phospholipid monolayer incorporating proteins such as perilipin 1a, perilipin 2, and Fsp27 [5]. Lipid droplet-related proteins, such as perilipin, comparative gene identification-58 (CGI-58), adipose triglyceride lipase (ATGL), and hormone sensitive lipase (HSL), and the way they interact with one another play important roles in regulating lipid accumulation and utilization [6–8]. At their basal expression levels, perilipin and CGI-58 limit the activity of ATGL, suppressing adipose tissue TAG decomposition to diacylglycerols (DAGs) [9]. Exercise-induced activation of catecholamine activates cAMP-dependent protein kinase A (PKA) [10], which phosphorylates proteins involved in lipolysis, such as perilipin and lipolytic enzymes [9]. HSL is attracted to perilipin in the cell membrane, promoting degradation of TAGs, DAGs, and monoacylglycerols to glycerols and fatty acids (FA) [9, 11].

When energy is needed, during starvation and exercise, stored TAGs are hydrolyzed by lipolysis to supply FA [12]. Exercise may promote lipolysis by stimulating the LD signaling pathway; exercise training, which induces fat loss by activating lipolysis in the adipose tissue, has become an accepted treatment for metabolic disorders.

Exercise is a preventive and therapeutic treatment for obesity because it activates lipolysis [13]. However, most people usually stop regular exercise for various reasons, thereby suffering from the detrimental effects of detraining [14]. Although it is obvious that regular and continuous exercise is necessary to maintain the positive changes induced by exercise, few studies have been conducted that examine the effect of detraining in combination with HFDs. Moreover, to our knowledge, the effects of regular exercise following a certain period of HFD have not been investigated.

Here we examined the effects of exercise on the expression levels of LD-related proteins and on visceral adipose tissue histology in mice with HFD-induced obesity. We also investigated the effects of detraining after regular exercise, as well as the effects of regular exercise after 15 weeks of HFD.

## Methods

### Animals

Seventy 4-week-old male C57BL/6 mice were used in this study. Three to four mice were housed per cage, and acclimatized for a week in the Dong-A University College of Medicine Animal Laboratory. The laboratory conditions were maintained constant: 55% relative humidity, 22 ± 2 °C and a 12-h dark-light cycle. The animal experiments were approved by the Dong-A University Medical School Institutional Animal Care and Use Committee (DIACUC-approval-14-5) and all procedures were conducted in accordance with the committee guidelines.

### Animal feed and obesity induction

Animals were randomly assigned to two groups: a normal diet + sedentary group (ND, *n* = 20) and a HFD + sedentary group (HF, *n* = 50). For six weeks, the HF group was fed with a 45% fat chow (45% lipid, 35% carbohydrate, and 20% protein) to induce obesity, whereas the ND was fed with a standard chow. All animals had free access to tap water. The animal body weight was measured every week.

### Exercise program

Immediately after the obesity induction period, the mice in the HF group were randomly subdivided into an HF (*n* = 30) and an HF + training (HFT, *n* = 20) groups. Following a one-week pre-exercise period for adaptation to maintaining a HFD, the animals in the HFT group underwent exercise training on a motor-driven animal treadmill five times per week for eight weeks. For the first four weeks of training, the exercise intensity was adjusted to 5 m/min for 5 min, 12 m/min for 30 min, and 5 m/min for 5 min, at 0% slope. For the last four weeks of training, the exercise intensity was increased to 5 m/min for 5 min, 14 m/min for 30 min, and 5 m/min for 5 min, also at 0% slope. This moderate intensity exercise protocol does not induce muscle damage. Ten mice in each group were sacrificed.

### Detraining and exercise after chronic HFD

**Fig. 1:**
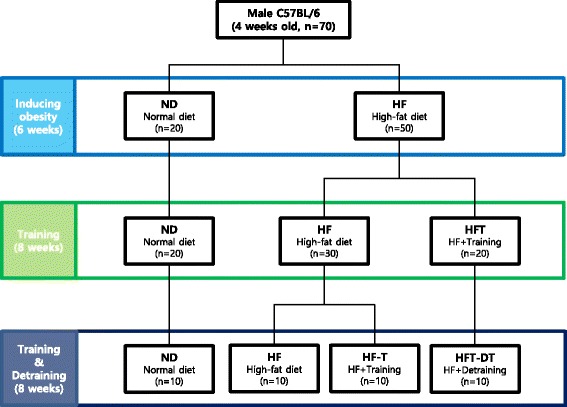
Experimental design. Experimental design of this study are presented. ND; Normal-diet + sedentary group, HF; High-fat diet + sedentary group, HFT; High-fat diet + Training group, HF-T; Training group after chronic HFD, HFT-DT; Detraining group after completion of regular exercise

To study the effects of detraining and exercise following chronic HFD, the mice in the HFT group stopped exercise after the eight-week exercise period (Detraining group; HFT-DT, *n* = 10), while, the mice in the HF group were randomly subdivided into two groups: HF (*n* = 10) and HF + training after chronic HFD (HF-T, *n* = 10); the animals in the HF and HF-T groups had ingested high-fat feeds for fifteen weeks without exercise (six weeks for inducing obesity; one week corresponding to the pre-exercise period of the HFT group; eight weeks corresponding to the exercise treatment of the HFT group). Exercise was carried out in the same manner as for the HFT group. The ten mice in each group were sacrificed 48 h after the completion of their last exercise, as for the HFT group. The experimental design of this study is presented in Fig. 1.

### Tissue sampling

In order to rule out temporary training effects, tissue sampling was conducted 48 h after the completion of the last exercise. Food was removed from the mouse cages 12 h prior to sacrifice. The animals were completely anesthetized with ethyl ether, and the abdominal visceral fat was excised. The samples were immediately weighed, frozen in liquid nitrogen, and stored at −80 °C.

### Analysis content

#### Western blot

To extract protein from the adipose tissue, the tissues were lysed in 200 μl radioimmunoprecipitation assay (RIPA) buffer. The tissue was homogenized and centrifuged for 30 min at 14,000 rpm. The protein concentration of the supernatant was measured using the BCA protein assay kit (PIERCE, USA). Samples of equal protein content were resolved by SDS-polyacrylamide gel electrophoresis on a 10 or 12% gel, and transferred to a membrane. The membrane was blocked with 5% skim milk in phosphate-buffered saline (PBS) (NaCl 8 g, KCI 0.2 g, Na_2_HPO_4_ 1.44 g, KH_2_PO_4_ 0.24 g, pH 7.4), and subsequently incubated at 4 °C overnight with primary antibodies (1:1000 dilution) against PKA (sc-98951), perilipin1 (sc-47320), CGI-58 (sc-100468), ATGL (sc-67355), and HSL (sc-25843) (all from Santa Cruz Biotechnology, USA). The membrane was incubated with goat anti-mouse or anti-rabbit IgG conjugated secondary antibody for 1 h at room temperature. The signal was developed with an ECL solution (Amersham Pharmacia Biotech, USA) and visualized with the ImageQuant™ LAS-4000 system (GE Healthcare, Sweden).

#### Histological analysis

Small pieces of abdominal visceral fat were fixed with formalin (10% neutral-buffer formalin), and embedded in paraffin. Five-micrometer sections were cut and stained using hematoxylin and eosin (H&E). Digital images of the slides were captured with an Aperio ScanScope (Aperio, USA).

### Statistical analysis

All statistical analysis was performed with the Statistical Package for Social Sciences (version 22.0); values are reported as means ± SE. To compare groups we performed analysis of variance (ANOVA), using the Duncan post hoc test to validate significant differences. A significance level of *P* = 0.05 was used as a threshold for statistical significance.

## Results

### Body weight changes

**Fig. 2:**
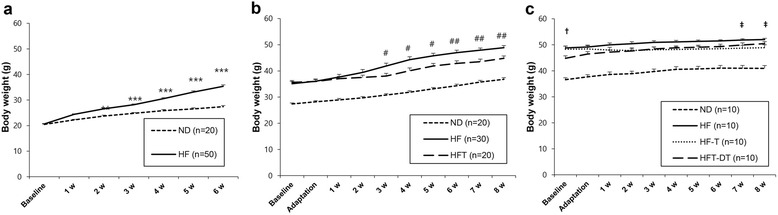
Body weight changes over time for each experimental group. Body weight changes during period of HFD to induce obesity (**a**), during 8 weeks of exercise treatment (**b**), and during 8 weeks of detraining treatment (**c**). Values are presented mean ± SE. ND; Normal-diet + sedentary group, HF; High-fat diet + sedentary group, HFT; High-fat diet + Training group, HF-T; Training group after chronic HFD, HFT-DT; Detraining group after completion of regular exercise. ^**^ *p* < 0.01, ^***^ *p* < 0.001; Significant difference between ND and HF group, ^#^ *p* < 0.05, ^##^ *p* < 0.01, ^###^ *p* < 0.001; Significant difference between HF and HFT group, ^†^ *p* < 0.05; Significant difference between HF and HFT-DT group. ^‡^ *p* < 0.05; Significant difference between HF and HF-T group

Body weight changes throughout the experimental period are presented in Fig. 2. There was a significant body weight difference between the ND and HF groups after two weeks of HFD (*P* < 0.01), which increased with time until the end of the obesity induction period (*P* < 0.001) (Fig. 2a). During the eight-week exercise period, a significant difference of body weight between the HF and HFT groups was observed after three weeks of exercise (*P* < 0.05), which increased with time until the end of the exercise period (*P* < 0.01) (Fig. 2b). Following eight weeks of detraining (Fig. 2c), the baseline body weight of the HFT-DT group was lower than that of the HF and HF-T groups (*P* < 0.05). However, after two weeks of detraining and until the end of the experiment, the body weight of the HFT-DT group was higher than that of the HF-T group. Moreover, a significant body weight difference between the HF-T and HF groups was observed after seven weeks of detraining.

### Abdominal visceral fat

**Fig. 3:**
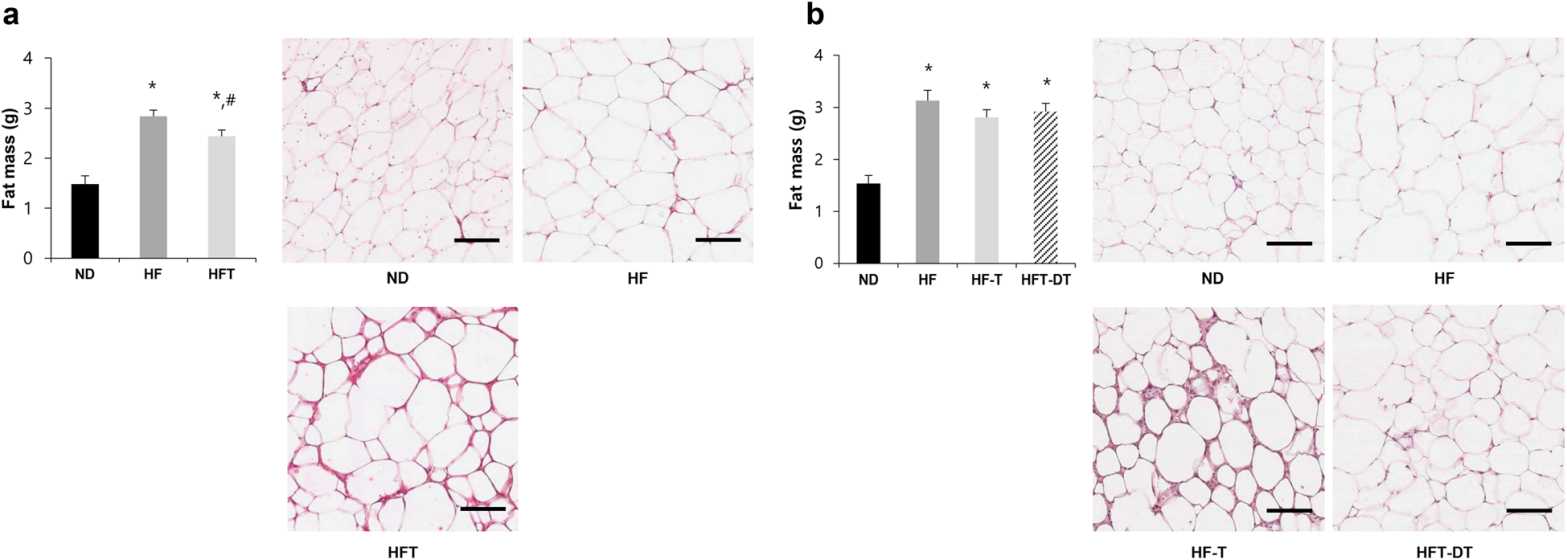
Abdominal visceral fat mass and histological changes after eight weeks of training and detraining. Fat mass and histological changes of adipose tissue after training (**a**) and detraining (**b**) are presented. Values are presented mean ± SE. ND; Normal-diet + sedentary group, HF; High-fat diet + sedentary group, HFT; High-fat diet + Training group, HF-T; Training group after chronic HFD, HFT-DT; Detraining group after completion of regular exercise. ^*^ *p* < 0.05; Significant difference with ND group, ^#^ *p* < 0.05; Significant difference with HF group. Scale bar = 100 μm

Abdominal visceral fat mass measurements and histological changes after eight weeks of training and detraining are presented in Fig. 3. After eight weeks of training, the abdominal visceral fat mass was significantly higher in the HF and HFT groups than in the ND group (*P* < 0.05), and significantly higher in the HF group than in the HFT group (*P* < 0.05). After eight weeks of detraining, the fat mass was higher in the HF, HF-T, and HFT-DT groups than in the ND group (*P* < 0.05), but a significant difference was not observed between the HF, HF-T, and HFT-DT groups.

### Protein levels

**Fig. 4:**
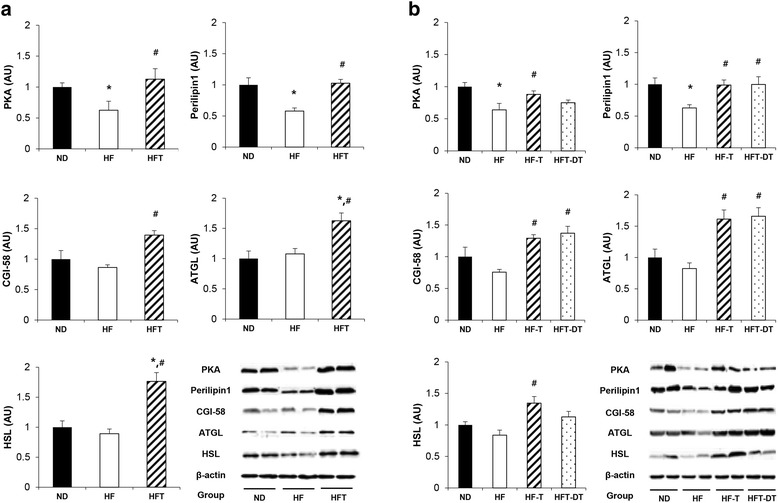
Expression level of proteins related to adipose tissue LD signaling pathway. Protein levels in the adipose tissue after training (**a**) and detraining (**b**) are presented. Values are presented mean ± SE. ND; Normal-diet + sedentary group, HF; High-fat diet + sedentary group, HFT; High-fat diet + Training group, HF-T; Training group after chronic HFD, HFT-DT; Detraining group after completion of regular exercise. ^*^ *p* < 0.05; Significant difference with ND group, ^#^ *p* < 0.05; Significant difference with HF group

Changes in the levels of proteins of the LD signaling pathway after eight weeks of training and detraining are presented in Fig. 4. After eight weeks of training, the levels of PKA and perilipin1 were significantly lower in the HF group than in the ND group (*P* < 0.05); in addition, PKA, perilipin1, and CGI-58 levels were significantly higher in the HFT group than in the HF group (*P* < 0.05). The levels of ATGL and HSL, key enzymes in lipolysis, were also higher in the HFT group than in the ND and HF groups (*P* < 0.05) (Fig. 4a).

Following eight weeks of detraining, the levels of PKA, perilipin1, and CGI-58 were significantly higher in the HF-T group than in the HF group (*P* < 0.05). The levels of perilipin1 and CGI-58 were significantly higher in the HFT-DT group than in the HF group (*P* < 0.05); however, a significant difference was not observed between the HF-T and HFT-DT groups. The levels of ATGL and HSL were significantly higher in the HF-T than in the HF group (*P* < 0.05); the ATGL level was significantly higher in the HFT-DT group than in the HF group (*P* < 0.05).

## Discussion

Chronic HFD increases the fat mass, which is closely related to metabolic disorders such as dyslipidemia, hypertension, insulin resistance, and type 2 diabetes [15]. Visceral fat in particular has been used as a powerful metabolic disease predictor, more than subcutaneous fat does [3, 16]. In this study, we found that abdominal visceral fat was reduced by regular exercise. However, the visceral fat mass was increased after detraining, indicating that the benefits from exercise can quickly disappear. We also found that regular exercise without caloric restriction increased the expression of LD-related proteins in the adipose tissue of obese mice, which was accompanied by reductions in visceral fat mass and LD size. Moreover, exercise after fifteen weeks of HFD increased LD-related protein levels and inhibited the increase of abdominal visceral fat mass, although detraining had the opposite effect.

Chronic HFD also increases body weight. Regular exercise and caloric restriction are effective treatments for the regulation of body weight [17, 18]. In this study, as expected, HFD increased fat mass and body weight, but regular exercise without a caloric restriction inhibited the increase in weight gain. Moreover, body weight did not increase by exercise after continuous HFD, similar to effect of exercise after short-term HFD. In contrast, the body weight of the detraining group increased during the detraining period, especially, at the early detraining period, suggesting that the greatest effect might occur during the initial stage of detraining. Therefore, detraining in combination with fat-rich food consumption can increase body weight, and continuing the HFD may rapidly lead to obesity.

Consumed fat is mainly stored as TAGs in LD. Perilipin, which is well known as an important regulator of LD lipolysis, has been suggested to act as a regulator of lipogenesis as well [11, 19]. In obesity, the levels of perilipin in adipocytes were decreased, resulting in reduced lipolytic rates [20]. In contrast, perilipin transgenic mice had reduced white adipose tissue and gained less weight than wild-type mice challenged with HFD, suggesting that increased expression of perilipin protects against diet-induced obesity [21]. Overexpression of perilipin in adipocytes is a potential strategy for treating obesity, as PPAR-γ coactivator-1-alpha (PGC-1α) was increased in the adipose tissue of perilipin transgenic mice [22]; PGC-1α is a regulator of uncoupling protein-1, the brown adipocyte specific protein implicated in beta-oxidation attenuation.

As mentioned above, it is well known that HFD leads to obesity through the decrease of proteins involved in lipolysis, and regular exercise protects against the metabolic disorder through the increase of LD-related proteins in the adipose tissue. However, the effects of detraining and training after continuous HFD have not been investigated. We hypothesized that regular exercise after long-term HFD might also promote lipolysis, similar to exercise after short-term HFD, but the positive effects induced by exercise might disappear by detraining.

In this study, as expected, HFD led to a decrease in the perilipin levels, whereas exercise significantly increased perilipin and CGI-58 protein levels. These results are consistent with those of previous studies, showing that expression of perilipin and CGI-58 increased upon treadmill training [23]. In addition, although obesity-induced oxidative stress reduced the protein levels of HSL and ATGL, it also reduced adipose tissue size through the upregulation of antioxidant enzymes and lipolytic metabolism [24].

Although it is well known that regular exercise is an effective treatment for obesity, most people usually quit regular exercise for various reasons. A previous study reported that swimming training improved heart function, but the effects disappeared after only two weeks of detraining [25]. Moreover, although BDNF and NGF expression levels in the brain were elevated after eight weeks of exercise, the positive effects disappeared after six weeks of detraining [26]. In this study, we showed that eight weeks of detraining decreased the levels of PKA, which is the starting point of LD decomposition, although perilipin and CGI-58 protein levels did not decrease. Moreover, HSL protein levels were reduced after detraining, whereas ATGL protein levels were not significantly different from those before exercise. A previous study reported that DAG decomposition is not caused by a decrease in HSL levels, although triacylglycerol decomposition is caused by an increase in ATGL levels [27]. Consistent with the results of this study, our results suggest that reduced HSL protein levels do not induce adipose tissue decomposition. On the other hand, here we showed that regular exercise after continuous HFD increased perilipin and CGI-58 protein levels, and inhibited body weight and fat mass increase; these inhibitory effects might be the result of the increased levels of lipolytic enzymes, such as ATGL and HSL, in abdominal visceral fat after regular exercise, despite the chronic HFD challenge. As we did not directly analyze the amount of lipolysis induced by LD-related proteins, the effects of increased LD-related proteins on lipolysis upregulation are still unclear. However, previous work demonstrated that increased LD-related proteins by various treatments resulted in increased lipolytic activity, as assessed by glycerol and FA release [12]. Therefore, the increased levels of LD-related proteins we observed in this study might be responsible for the reduced fat mass and size, explaining why regular exercise amplifies lipolysis.

## Conclusion

To conclude, chronic HFD increases body weight and fat mass, accompanied by a decrease in the levels of LD-related proteins. Body weight and visceral fat mass were also increased after detraining, indicating that the benefits from exercise can quickly disappear. However, regardless of dietary restrictions, exercise is an effective treatment for obesity, owing to the regulation of LD signaling proteins. Moreover, the effects of regular exercise in the presence of HFD and after chronic HFD are similar to those of exercise in the absence of HFD. Therefore, although obesity is induced by chronic HFD, exercise without dietary change is sufficiently effective for obesity treatment regardless of the preceding HFD period.

## Abbreviations

ATGL: Adipose triglyceride lipase
CGI-58: Comparative gene identification-58
DAGs: Diacylglycerols
FA: Fatty acids
HFD: high-fat diet
HSL: Hormone sensitive lipase
LDs: Lipid droplets
PKA: cAMP-dependent protein kinase A
TAG: Triacylglyceride
